# Biochar derived from olive oil pomace mitigates salt stress on seedling growth of forage pea

**DOI:** 10.3389/fpls.2024.1398846

**Published:** 2024-08-19

**Authors:** Mehmet Kerim Gullap, Tuba Karabacak, Sedat Severoglu, Ayse Nida Kurt, Melek Ekinci, Metin Turan, Halit Aktas, Ertan Yildirim

**Affiliations:** ^1^ Department of Field Crops, Ataturk University, Erzurum, Türkiye; ^2^ Department of Horticulture, Ataturk University, Erzurum, Türkiye; ^3^ Faculty of Economy and Administrative Sciences, Department of Agricultural Trade and Management, Yeditepe University, Istanbul, Türkiye

**Keywords:** biochar, forage crops, plant growth, PEA, salinity

## Abstract

Studies are being conducted to develop strategies to reduce the adverse effects of salinity stress. In the present study, it was aimed to determine the interactive effects of salinity stress with biochar on plant growth—the physiological and biochemical attributes of forage peas (*Pisum sativum* ssp. *arvense* L.). Salt applications were carried out with irrigation water at concentrations of 0, 25, 50, 75, and 100 mM NaCl. The experiment was conducted using a randomized complete block design with three applications [control: 0 (B_0_), 2.5% biochar (B_1_), and 5% biochar (B_2_)], five salt doses [0 (S_0_), 25 (S_1_), 50 (S_2_), 75 (S_3_), and 100 (S_4_) mM NaCl], and three replications, arranged in a 3 × 5 factorial arrangement. In the salt-stressed environment, the highest plant height (18.75 cm) and stem diameter (1.71 mm) in forage pea seedlings were obtained with the application of B_1_. The root fresh (0.59 g/plant) and dry weight (0.36 g/plant) were determined to be the highest in the B_1_ application, both in non-saline and saline environments. A decrease in plant chlorophyll content in forage pea plants was observed parallel to the increasing salt levels. Specifically, lower H_2_O_2_, MDA, and proline content were determined at all salt levels with biochar applications, while in the B_0_ application these values were recorded at the highest levels. Furthermore, in the study, it was observed that the CAT, POD, and SOD enzyme activities were at their lowest levels at all salt levels with the biochar application, while in the B_0_ application, these values were determined to be at the highest levels. There was a significant decrease in plant mineral content, excluding Cl and Na, parallel to the increasing salt levels. The findings of the study indicate that biochar amendment can enhance forage peas’ growth by modulating the plant physiology and biochemistry under salt stress. Considering the plant growth parameters, no significant difference was detected between 2.5% and 5% biochar application. Therefore, application of 2.5 biochar may be recommended.

## 1 Introduction

The provision of healthy food, a necessity for the rapidly growing world population, will be one of the foremost challenges in the future. To meet the nutritional needs of the increasing population, both plant and animal production capacities need to be doubled (Howell et al., 2001). However, salinity, referred to as “white death,” stands among the most critical issues affecting global soils (Taha et al., 2020; Dustgeer et al., 2021; Ahmad et al., 2023a). Millions of hectares of land are damaged every year due to excessive salinity, and significant agricultural losses occur worldwide (Singh, 2022). Dry and semi-arid areas worldwide constitute approximately 46% of the total land area, with observed salinity issues in about 50% of irrigated lands in these regions. Saline soils cover around 7% of the Earth’s total land area (Ruiz-Lozano et al., 2001), and about 5% of the cultivated land, equivalent to around 77 million hectares out of the approximately 15 billion hectares processed, is affected by excessive salinity (Sheng et al., 2008). Salinity, a limiting factor in increasing plant productivity and agricultural production in areas dedicated to agricultural activities, is predominantly prevalent in arid and semi-arid regions with low rainfall, high temperatures, and various influencing factors such as low precipitation, high evapotranspiration, elevated groundwater levels, saline irrigation water, and improper irrigation practices (Tietel et al., 2019). This situation poses a significant threat to food security, particularly for communities whose livelihoods depend on agricultural and livestock production (Cowiea et al., 2018).

Soil salinity significantly reduces crop yield as it diminishes the plant’s transpiration, respiration, water uptake, and root development (Alkharabsheh et al., 2021; Bhattacharya, 2021; Abdelaal et al., 2022). Salinity stress, a significant constraining environmental factor, imposes a restrictive effect of 20% or more on plant growth and development (Porcel et al., 2012). Many economically important crops are sensitive to salinity, struggling to thrive under salty conditions and experiencing substantial decreases in yield (Wang et al., 2023; Ahmad et al., 2023b). In soils where many cultivated forage crops are grown, the presence of salt at levels limiting their growth and development leads to significant reductions in productivity. Particularly in areas where forage crops are cultivated in arid and semi-arid regions, saline soils impose limitations on yield (Ates and Tekeli, 2007).

Forage legumes, which are among the primary sources of roughage meeting the nutritional needs of animals and are rich in crude protein, vitamins, and minerals, are generally highly sensitive to salinity (Yavas and Unay, 2018). Salinity affects nodule formation in the roots of forage legume, either directly or indirectly, by reducing nodulation (Hanumantha et al., 2016). In the classification based on the salt tolerance of forage crops, the common forage pea (*Pisum sativum* ssp. *arvense* L.) is classified among salt-sensitive forage crops (Kayin et al., 2022, 2024).

Recently, biochar (BC) has gained significant interest in soil improvement. This carbon-rich material is produced by heating waste organic matter, like crop leftovers, in a low-oxygen environment. Biochar’s benefits for soil are numerous, including increased crop yields, better nutrient retention, improved soil structure, and contributions to waste management and carbon capture (Zhang et al., 2020). Produced from agricultural waste, biochar shows promise for environmental cleanup. This is due to its beneficial properties, including high heat resistance, a large surface area, varied pore sizes, and the presence of various functional groups. Additionally, biochar derived from agricultural waste can improve soil fertility and effectively capture contaminants from gas, during composting, and in other processes like anaerobic digestion and catalysis. Agricultural biomass stands out as a perfect choice for creating porous carbon materials. Its advantages are plentiful: readily available, cost-effective, renewable, rich in carbon, and environmentally friendly (Khedulkar et al., 2023).

Turkey yields most of the world’s olive oil production with an annual amount of 420,000 tons in the recent years. Olive pomace is a by-product consisting of the oil, pit, peel, and pulp remaining after the oil is extracted from olives. The pulp remaining after the oil is extracted, called “pomace”, is used in different sectors. Pomace is a residue of olive oil factories and is an important type of biomass seen in Mediterranean countries. Pomace can be obtained in quite large quantities at low cost. The olive oil pomace can provide a very important agricultural product, although it is currently wastefully incinerated for heating or left on the ground for slow decay. Alternatively, olive oil pomace represents a valuable source of carbon and can be manipulated to furnish the soil with high-quality organic matter in the form of biochar (Ainane et al., 2023; Kavdir et al., 2023). The ingredients in a plant’s growing environment significantly impact how well seeds germinate and the overall health of the seedlings. The medium directly affects germination, growth, and development, all the way down to influencing the future health of the root system. A good growing medium provides the necessary support for the plant to stand upright, stores nutrients and water for the plant to use, allows oxygen to reach the roots, and enables the roots to exchange gases with the air around them. Biochar has gained considerable importance in the improvement of degraded agricultural areas, waste management, and renewable energy production (Enaime and Lubken, 2021). Biochar is a carbon-rich organic material formed by the pyrolysis of plant wastes in an oxygen-free or low-oxygen environment (Gupta et al., 2022). It is suggested that biochar, which is widely used as a soil amendment to alleviate these negative effects and is actively employed in many parts of the world, with its extensive surface area, could be utilized under salt stress conditions (Ortas, 2016; Xiao et al., 2016; Ali et al., 2017). In conclusion, the use of biochar can reduce the impact of soil salinity on plants, promoting healthy plant development. Additionally, biochar application has the potential to enhance plant tolerance to salt stress by increasing photosynthetic productivity and overall plant growth (Kul et al., 2021a; Yildirim et al., 2022). Quality seedlings are a high-value product that can improve the early establishment of crops; increase finish crop quality, uniformity, and yield; and decrease production time. To our best knowledge, there is limited information found in the literature in terms of examining the effect of biochar obtained from olive oil pomace on seedling growth in forage peas grown under salt stress. In this context, the aim of this study was to enhance the development, growth, nutrient uptake, and salt tolerance of salt-sensitive forage peas (*Pisum sativum* ssp. *arvense* L.) through the application of biochar derived from olive oil pomace under salt stress conditions.

## 2 Materials and methods

The study was conducted in the greenhouses of Atatürk University, Plant Production Application and Research Center, as well as in the laboratories of the Department of Field Crops and the Department of Horticulture at the Faculty of Agriculture. Forage pea (*Pisum sativum* ssp. *arvense* L. cv Taşkent) seeds were used as the experimental material in the research. In the study, there were three biochar [B_0_: without biochar (control), B_1_: 2.5% biochar, and B_2_: 5% biochar] and five salinity doses (S_0_: 0, S_1_: 25 mM, S_2_: 50 mM, S_3_: 75 mM, and S_4_: 100 mM NaCl) in 15 different combinations (T_0_: B_0_S_0_, T_1_: B_0_S_1_, T_2_: B_0_S_2_, T_3_: B_0_S_3_, T_4_: B_0_S_4_, T_5_: B_1_S_0_, T_6_: B_1_S_1_, T_7_: B_1_S_2_, T_8_: B_1_S_3_, T_9_: B_1_S_4_, T_10_: B_2_S_0_, T_11_: B_2_S_1_, T_12_: B_2_S_2_, T_13_: B_2_S_3_, and T_14_: B_2_S_4_) in a 3 × 5 factorial design with three replications. The properties of the soil used in the experiment are given in **Table 1**.

**Table 1:** Characteristics of the soil used in the study.

Contents	Values	Contents	Values
pH	7.09 ± 0.17	Mg (cmol/kg)	11.99 ± 0.52
EC (μmhos/cm)	81.45 ± 2.81	Na (cmol/kg)	1.16 ± 0.07
CaCO_3_ (%)	1.31 ± 0.16	B (ppm)	0.02 ± 0.002
OM (%)	0.63 ± 0.06	Cu (ppm)	0.11 ± 0.006
Total N (%)	0.03 ± 0.002	Fe (ppm)	0.59 ± 0.35
P (ppm)	33.55 ± 2.55	Zn (ppm)	0.03 ± 0.001
K (cmol/kg)	2.21 ± 0.03	Mn (ppm)	0.15 ± 0.04
Ca (cmol/kg)	11.43 ± 0.28	Cl (ppm)	2.59 ± 0.27

The properties of the biochar used in the present study are provided in **Table 2**. In this research, the biochar was originated from olive oil pomace. To create the biochar, a special reactor was used to slowly pyrolyze the shells at a temperature of 550°C. The biochar used in the study was produced using the thermal conversion process. This process consists of three stages: first stage reactor (depolymerization), second stage reactor (hydrolysis), and third stage reactor (cracker). The concenter solid intermediate transported to this reactor was broken down into short hydrocarbon chains at a high temperature (550°C), from which energy products (renewable natural gas and renewable crude oil) and biochar were obtained.

**Table 2:** Characteristics of the biochar used in the study.

Contents	Values	Contents	Values
**OM (%)**	23.70	Na (ppm)	199
**CaCO_3_ (%)**	4.94	P (ppm)	110
**pH**	7.88	Fe (ppm)	37.04
**EC (mS/cm)**	2.34	Mn (ppm)	7.43
**Ca (ppm)**	14.564	Zn (ppm)	21.12
**Mg (ppm)**	905	Cu (ppm)	23.86
**K (ppm)**	517		

In the study, biochar application was carried out in containers (dimensions: 720 × 195 × 155 mm) with a soil mixture of soil, sand, and peat (3:1:1, v/v/v). Three doses were applied: 0% (control), 2.5%, and 5% of the soil weight.

In the prepared pots, 40 seeds were planted and salt applications were carried out with irrigation water at concentrations of 0, 25, 50, 75, and 100 mM NaCl after seed sowing. To prevent immediate salt stress damage to the seeds, the salt concentration started at 25 mM and was gradually increased, finally reaching the predetermined maximum doses. Salt application was performed by controlling the soil moisture and EC values in the form of irrigation water.

Plants were harvested after 50 days from sowing, where chlorophyll reading value, plant height, stem diameter, and fresh weights of plant and root were determined. Dry weights were determined after drying of fresh plant material in an oven at 67°C for 48 h. Fresh leaf samples were kept at −80°C for analyses.

Leaf area was determined with a CI-202 portable area meter (CID, Inc., USA).

To determine the leaf chlorophyll concentration of plants, samples were cut at 10-mm diameter from the middle leaves and put into 2-mL Eppendorf tubes. These discs were then placed in tubes containing cold acetone and shaken for 3 min. Next, the mixture was spun in a centrifuge to separate the chlorophyll extract. Finally, a device called a spectrophotometer (Thermo Scientific™ Multiskan™ FC Microplate Photometer) was used to measure the intensity of light absorbed by the chlorophyll extract at specific wavelengths (663 and 645 nm). Chlorophyll a, chlorophyll b, and total chlorophyll (mg g^−1^) were determined according to Lichtenthaler and Wellburm (1983).

The contents of P, K, Ca, Mg, S, Mn, Fe, Zn, B, Cl, Na, and Cu in the leaf and root samples were determined by using Optima 2100 DV, ICP/OES, Perkin-Elmer, Shelton, CT spectrophotometer. The nitrogen (N) content was determined according to the Micro Kjeldahl wet combustion method (Bremner, 1996; Dursun et al., 2010). The properties of the starting soil sample (pH, organic matter, EC, CaCO_3_, and mineral content) analyzed were determined by McLean (1982), Nelson and Sommers (1982), and Rhoades (1982).

The extraction and purification procedures for hormone analysis were conducted according to the method of Battal and Tileklioglu (2001). Indoleacetic acid (IAA), abscisic acid (ABA), gibberellic acid (GA), salicylic acid (SA), cytokinin, zeatin, and jasmonic acid were analyzed by using high-performance liquid chromatography (HPLC) through a Zorbax Eclipse-AAA C-18 column (Agilent 1200 HPLC) coupled with a UV detector of absorbance at 265 nm.

Lipid peroxidation was estimated as the malondialdehyde (MDA) content of leaves according to Heath and Packer (1968). H_2_O_2_ was determined according to Ohkawa et al. (1979).

The MDA content was assessed as the extent of lipid peroxidation in frozen leaves. The process involved grinding a 0.2-g leaf sample into powder using liquid nitrogen. The resulting mixture was centrifuged at 12,000 *g* for 20 min to obtain a clearer solution. To determine the MDA levels, a specific solution containing trichloroacetic acid (TCA), thiobarbituric acid (TBA), and an antioxidant (butylated hydroxytoluene) was mixed with a portion of the extract and heated. The reaction was then rapidly stopped by cooling the mixture in an ice bath. After centrifugation, the amount of light absorbed by the solution was measured at three wavelengths (400, 500, and 600 nm). Higher absorbance indicates more MDA, a breakdown product of lipids caused by peroxidation. A standard conversion factor (extinction coefficient of 155 mmol/L*cm) was used to calculate the final MDA concentration (Shams et al., 2019).

Superoxide dismutase (SOD) activity was determined by the spectrophotometric measurement of photochemical reduction of nitro blue tetrazolium (NBT). Peroxidase (POD) activity determination was based on the observation of absorbance increase at 470 nm caused by the colored compound, which is the product of the reaction in which guaiacol and H_2_O_2_ are substrates. The catalase (CAT) activity was measured based on the rate of H_2_O_2_ decomposition. The method applied by Liu et al. (2014) was used to determine the CAT, POD, and SOD activity, respectively.

A 50-mg frozen leaf sample was powdered with liquid nitrogen and extracted with a pestle and mortar with 4.5 mL of 5-sulfosalicylic acid 3% in an ice bath. The homogenates were filtered with a filter paper (#2). Then, 2 mL of filtrate was reacted with 2 mL acid-ninhydrin and 2 mL of glacial acetic acid in a test tube for 1 h at 100°C, and the reaction was terminated in an ice bath. The filtrates were used for the analysis. Proline concentration was assayed spectrophotometrically at 520 nm (Bates et al., 1973).

The experiment was established by using three replications at five pots in each replication, with two factors (salinity and biochar levels), in completely random design (CRD). For statistical analysis, an ANOVA (two-way ANOVA) technique for completely randomized design was carried out using SPSS 20.0 (SPSS 140 Inc.), and the mean differences were compared by using Duncan’s multiple-range test.

## 3 Results

### 3.1 Growth parameters

**Figure 1:**
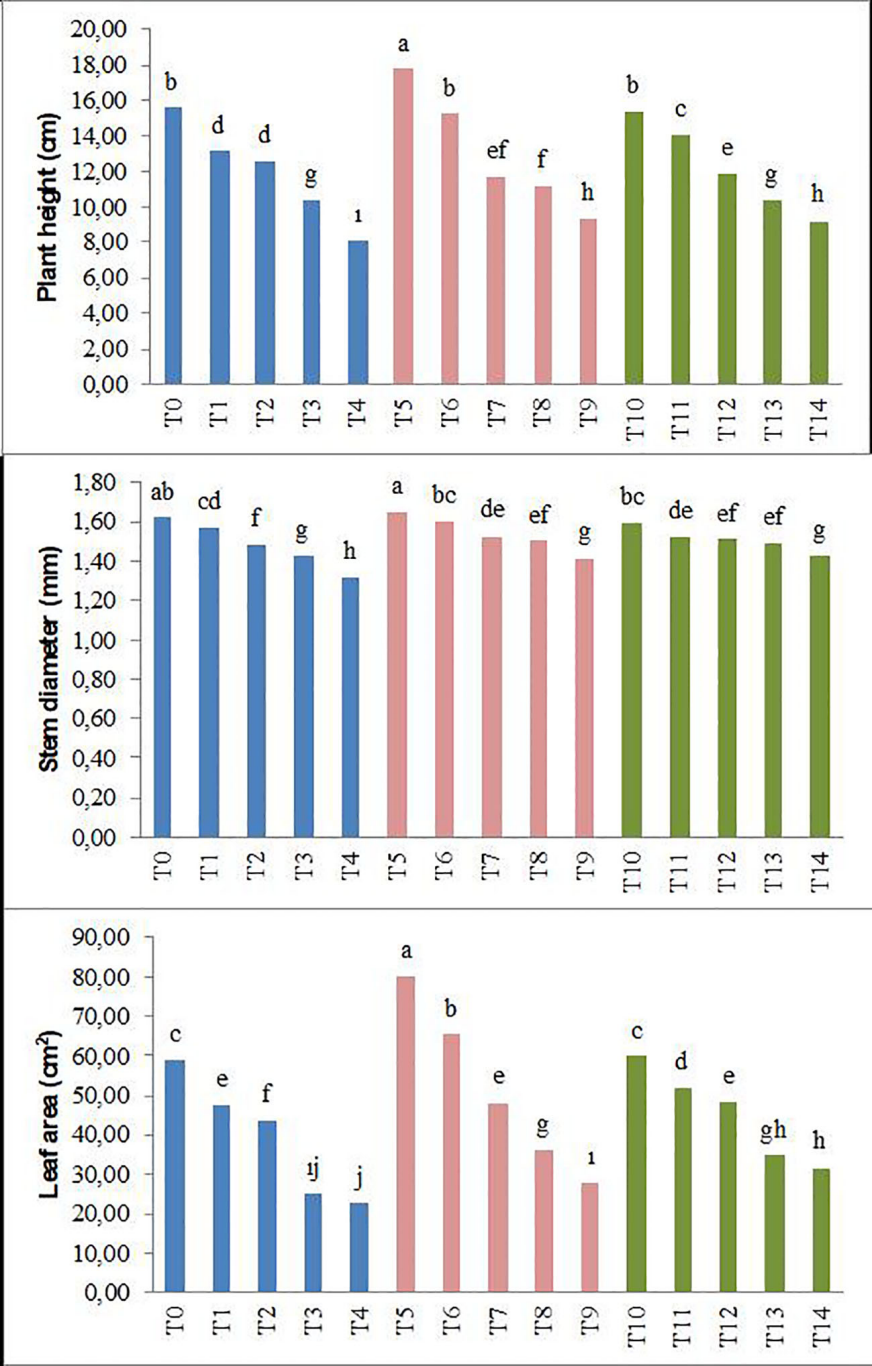
Effect of biochar on the plant height, stem diameter, and leaf area of forage pea seedlings under saline conditions. The different-lettered averages in each bar are significantly different according to Duncan’s multiple-range test (*p* ≤ 0.001). B_0_, without biochar (control); B_1_, 2.5% biochar; B_2_, 5% biochar; S_0_, 0 mM NaCl; S_1_, 25 mM NaCl; S_2_, 50 mM NaCl; S_3_, 75 mM NaCl; S_4_, 100 mM NaCl; T_0_, B_0_S_0_; T_1_, B_0_S_1_; T_2_, B_0_S_2_; T_3_, B_0_S_3_; T_4_, B_0_S_4_; T_5_, B_1_S_0_; T_6_, B_1_S_1_; T_7_, B_1_S_2_; T_8_, B_1_S_3_; T_9_, B_1_S_4_; T_10_, B_2_S_0_; T_11_, B_2_S_1_; T_12_, B_2_S_2_; T_13_, B_2_S_3_; T_14_, B_2_S_4._.

**Figure 2:**
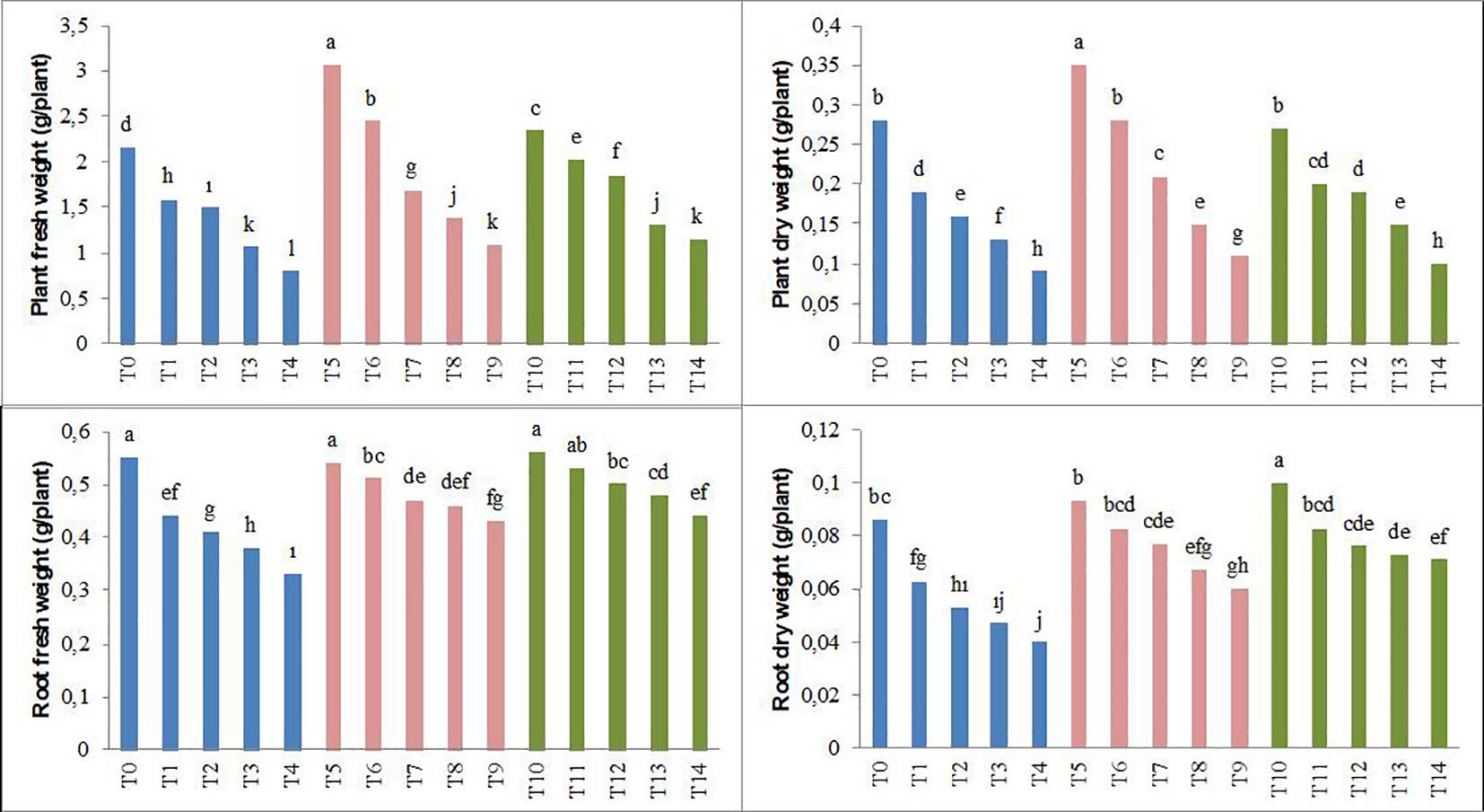
Effect of biochar on the plant fresh weight, plant dry weight, root fresh weight, and root dry weight of forage pea seedlings under saline conditions. The different-lettered averages in each bar are significantly different according to Duncan’s multiple-range test (*p* ≤ 0.001). B_0_, without biochar (control); B_1_, 2.5% biochar; B_2_, 5% biochar; S_0_, 0 mM NaCl; S_1_, 25 mM NaCl; S_2_, 50 mM NaCl; S_3_, 75 mM NaCl; S_4_, 100 mM NaCl; T_0_, B_0_S_0_; T_1_, B_0_S_1_; T_2_, B_0_S_2_; T_3_, B_0_S_3_; T_4_, B_0_S_4_; T_5_, B_1_S_0_; T_6_, B_1_S_1_; T_7_, B_1_S_2_; T_8_, B_1_S_3_; T_9_, B_1_S_4_; T_10_, B_2_S_0_; T_11_, B_2_S_1_; T_12_, B_2_S_2_; T_13_, B_2_S_3_; T_14_, B_2_S_4._.

The results obtained in the study, as shown in **Figures 1**, **2**, indicate that the application of biochar to forage peas reduces the adverse effects of salt stress on the plants.

Parameters such as plant height, stem diameter, leaf area, plant fresh weight, root fresh weight, plant dry weight, and root dry weight showed significant decreases in forage pea seedlings with the increase in salinity levels compared to the control groups. This decrease was 48% (plant height), 19% (stem diameter), 62% (leaf area), 63% (plant fresh weight), 40% (root fresh weight), 68% (plant dry weight), and 53% (root dry weight), respectively. However, the application of biochar derived from olive oil pomace has positively mitigated the adverse effects of salinity stress on the examined parameters. In the study, particularly in the saline environment, the highest plant height and stem diameter were achieved with the biochar applications (**Figures 1**, **2**).

Additionally, according to the average data, the T_5_ application of biochar resulted in the highest plant fresh and dry weight, while the T_10_ application led to the highest root fresh and dry weight. The decrease caused by the highest salt level in plant fresh and dry weight and root fresh and dry weight was lower in the B_2_ application (T_14_). In conclusion, the study determined significant differences between salinity and applied biochar for all of the growth parameters examined. Considering the plant growth parameters, no significant difference was detected between 2.5% and 5% biochar application.

### 3.2 Chlorophyll content and SPAD value

**Figure 3:**
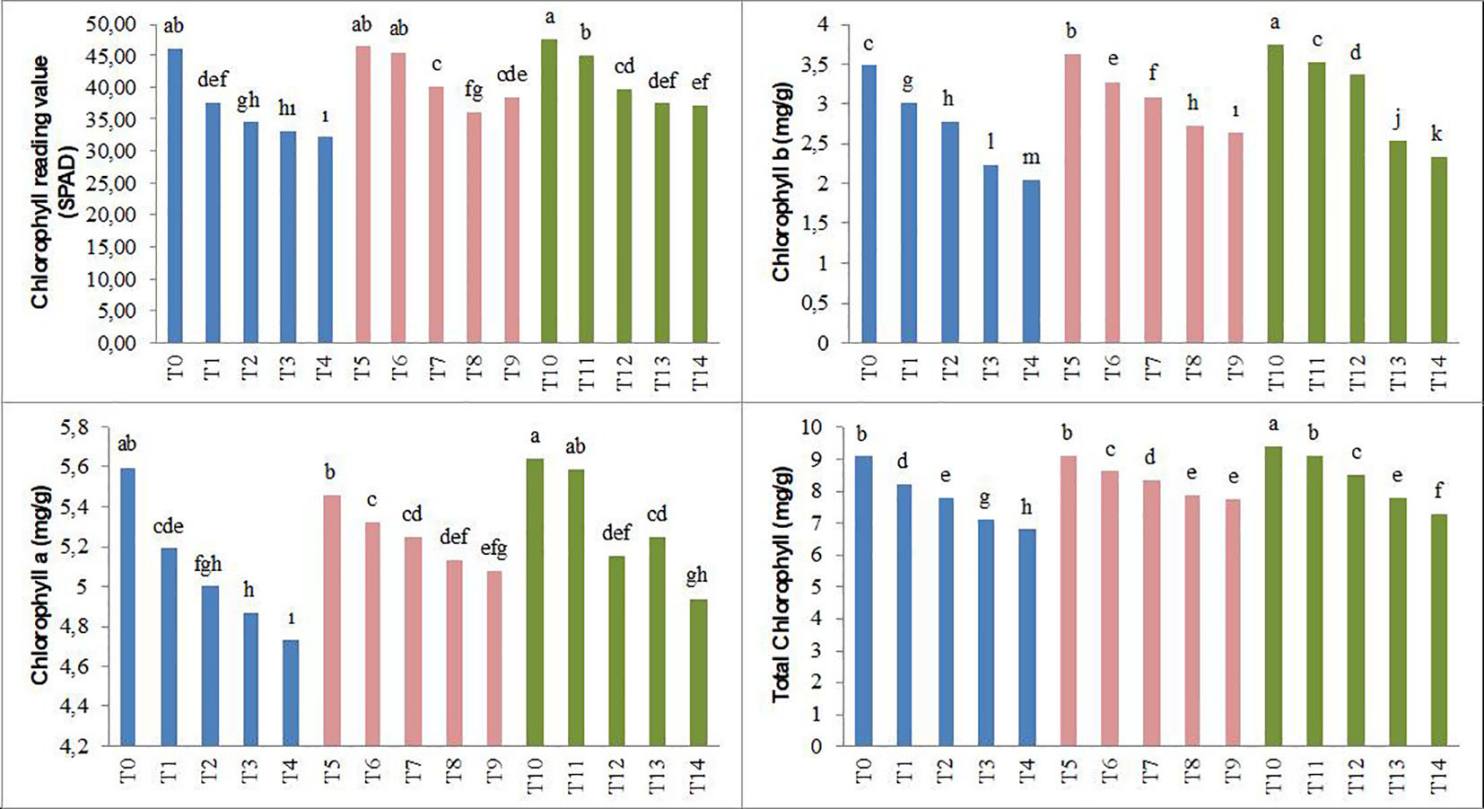
Effect of biochar on the chlorophyll reading value and chlorophyll content of forage pea seedlings under saline conditions. The different-lettered averages in each bar are significantly different according to Duncan’s multiple-range test (*p* ≤ 0.001). B_0_, without biochar (control); B_1_, 2.5% biochar; B_2_, 5% biochar; S_0_, 0 mM NaCl; S_1_, 25 mM NaCl; S_2_, 50 mM NaCl; S_3_, 75 mM NaCl; S_4_, 100 mM NaCl; T_0_, B_0_S_0_; T_1_, B_0_S_1_; T_2_, B_0_S_2_; T_3_, B_0_S_3_; T_4_, B_0_S_4_; T_5_, B_1_S_0_; T_6_, B_1_S_1_; T_7_, B_1_S_2_; T_8_, B_1_S_3_; T_9_, B_1_S_4_; T_10_, B_2_S_0_; T_11_, B_2_S_1_; T_12_, B_2_S_2_; T_13_, B_2_S_3_; T_14_, B_2_S_4._.

The results of the study revealed that, compared to the control conditions, under saline conditions (25, 50, 75, and 100 mM NaCl), SPAD, chlorophyll a, chlorophyll b, and total chlorophyll values in forage pea seedlings decreased by 18%, 25%, 28%, and 30%; 7%, 11%, 13%, and 16%; 13%, 20%, 36%, and 41%; and 9%, 14%, 22%, and 25%, respectively. The T_5_–T_9_ and T_10_–T_14_ biochar applications showed an increasing trend in the parameters that tended to decrease under increasing salinity conditions (**Figure 3**).

### 3.3 H_2_O_2_, MDA, and proline contents

**Figure 4:**
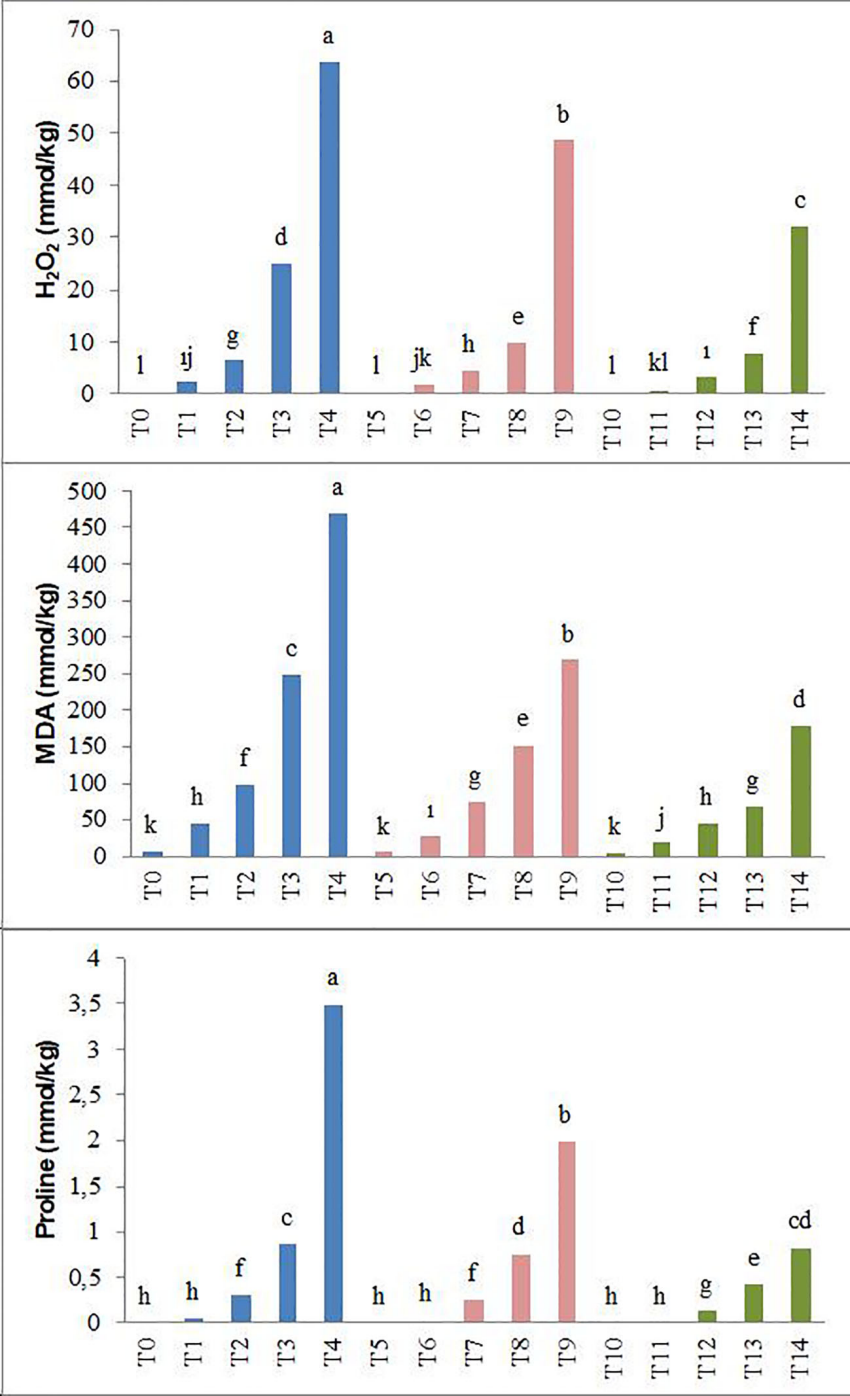
Effect of biochar on H_2_O_2_, MDA, and proline content of forage pea seedlings under saline conditions. The different-lettered averages in each bar are significantly different according to Duncan’s multiple-range test (*p* ≤ 0.001). B_0_, without biochar (control); B_1_, 2.5% biochar; B_2_, 5% biochar; S_0_, 0 mM NaCl; S_1_, 25 mM NaCl; S_2_, 50 mM NaCl; S_3_, 75 mM NaCl; S_4_, 100 mM NaCl; T_0_, B_0_S_0_; T_1_, B_0_S_1_; T_2_, B_0_S_2_; T_3_, B_0_S_3_; T_4_, B_0_S_4_; T_5_, B_1_S_0_; T_6_, B_1_S_1_; T_7_, B_1_S_2_; T_8_, B_1_S_3_; T_9_, B_1_S_4_; T_10_, B_2_S_0_; T_11_, B_2_S_1_; T_12_, B_2_S_2_; T_13_, B_2_S_3_; T_14_, B_2_S_4._.

It has been observed that the levels of hydrogen peroxide (H_2_O_2_), malondialdehyde (MDA), and proline in the plant significantly increased under different salinity conditions (25, 50, 75, and 100 mM NaCl) compared to the control. However, both levels of biochar application (T_5_–T_9_ and T_10_–T_14_) have been recorded to reduce the H_2_O_2_, MDA, and proline content in forage pea seedlings under salinity stress. Especially the T_10_–T_14_ application resulted in lower H_2_O_2_, MDA, and proline content at all salt levels, while in the T_0_–T_4_ application these values were determined to be the highest (**Figure 4**).

### 3.4 Antioxidant enzyme activity

**Figure 5:**
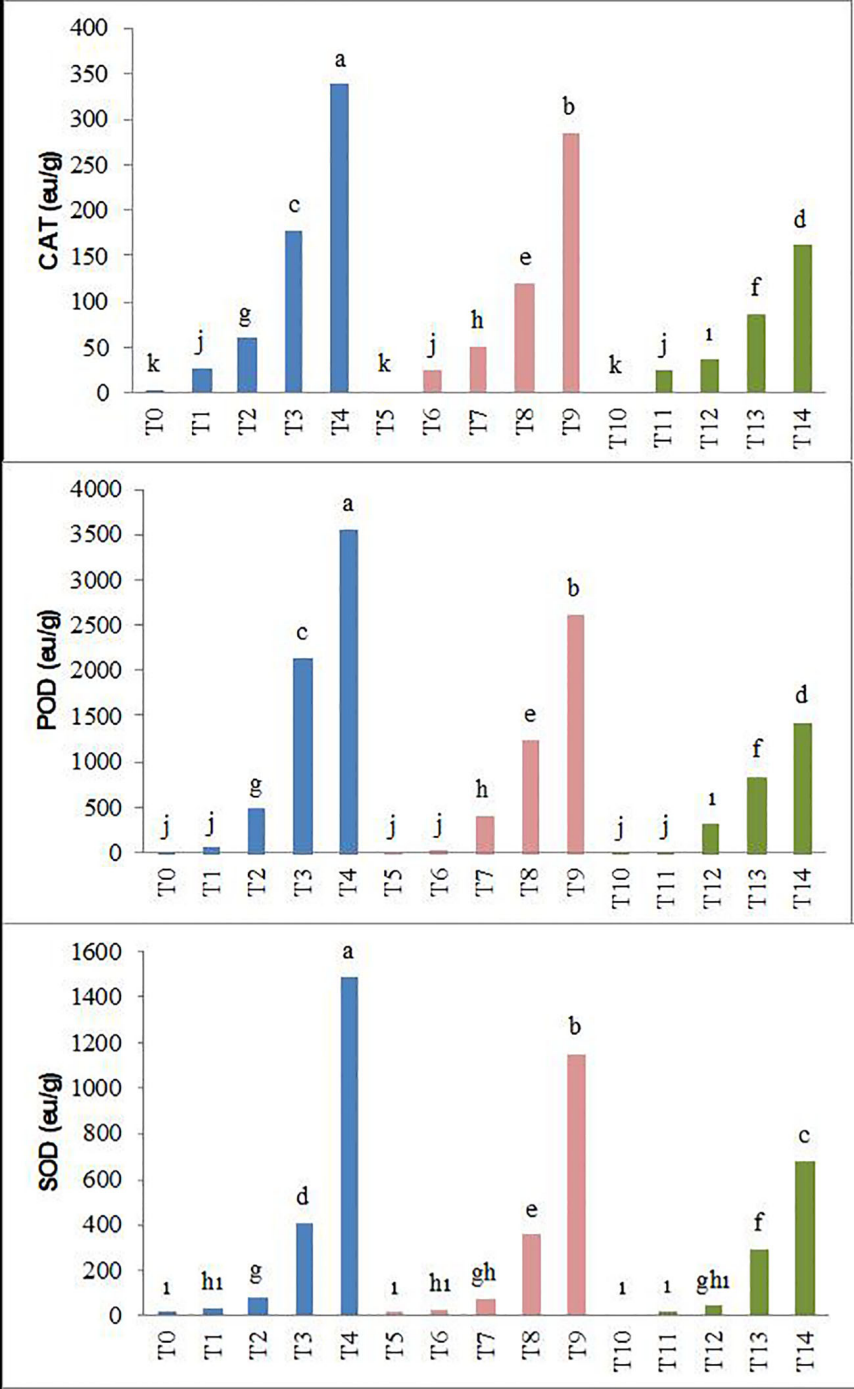
Effect of biochar on CAT, POD, and SOD enzyme activity of forage pea seedlings under saline conditions. The different-lettered averages in each bar are significantly different according to Duncan’s multiple-range test (*p* ≤ 0.001). B_0_, without biochar (control); B_1_, 2.5% biochar; B_2_, 5% biochar; S_0_, 0 mM NaCl; S_1_, 25 mM NaCl; S_2_, 50 mM NaCl; S_3_, 75 mM NaCl; S_4_, 100 mM NaCl; T_0_, B_0_S_0_; T_1_, B_0_S_1_; T_2_, B_0_S_2_; T_3_, B_0_S_3_; T_4_, B_0_S_4_; T_5_, B_1_S_0_; T_6_, B_1_S_1_; T_7_, B_1_S_2_; T_8_, B_1_S_3_; T_9_, B_1_S_4_; T_10_, B_2_S_0_; T_11_, B_2_S_1_; T_12_, B_2_S_2_; T_13_, B_2_S_3_; T_14_, B_2_S_4_.

In parallel with the increasing salinity, statistically significant increases have occurred in the enzyme activities of CAT, POD, and SOD in pea seedlings. The increase caused by salinity in these parameters was lower with biochar applications. Specifically, the T_10_–T_14_ application resulted in the lowest CAT, POD, and SOD enzyme activities at all salt levels, while the T_0_–T_4_ application showed the highest levels (**Figure 5**).

### 3.5 Hormones

The content of indole-3-acetic acid (IAA), gibberellic acid (GA), salicylic acid (SA), cytokinin, zeatin, and jasmonic acid in forage pea seedlings, except for abscisic acid (ABA), has shown a significant decrease in response to the increased salinity levels. In general, biochar applications have reduced the content of IAA, GA, SA, cytokinin, zeatin, and jasmonic acid hormones in forage pea seedlings below normal conditions compared to the control, excluding ABA content (**Table 3**).

**Table 3:** Effect of biochar on the content of IAA, ABA, GA, SA, cytokinin, zeatin, and jasmonic acid in forage pea seedlings under saline conditions.

Treatments	IAA, ng/mg tissue	ABA, ng/g DW	GA, ng/gDW	SA, ng/g DW	Cytokinin, ng/g DW	Zeatin, ng/g DW	Jasmonic acid, ng/gDW
T_0_	2.30 c	17.97 j	2.57 c	58.36 c	52.04 c	23.05 ab	70.71 c
T_1_	1.82 d	59.69 j	1.97 e	18.28 f	24.16 f	8.92 c	39.94 e
T_2_	0.62 f	390.70 g	1.03 gh	7.65 g	4.90 i	3.08 c	7.30 h
T_3_	0.24 hi	1,134.21 d	0.62 i	4.03 h	2.37 j	1.26 c	3.09 ij
T_4_	0.05 j	5,321.18 a	0.15 k	0.91 h	0.34 j	0.38 c	1.12 j
T_5_	4.42 a	13.88 j	2.90 b	77.89 b	77.03 b	29.69 a	88.43 b
T_6_	1.91 d	39.17 j	2.16 d	26.41 e	35.27 e	30.85 a	57.70 d
T_7_	0.70 f	260.69 h	1.13 g	10.88 g	9.60 h	6.01 c	13.37 g
T_8_	0.29 gh	942.99 e	0.53 ij	7.85 g	4.74 i	2.14 c	6.34 hi
T_9_	0.10 ij	3,606.06 b	0.18 k	1.69 h	0.70 j	0.67 c	1.74 j
T_10_	2.78 b	7.58 j	3.75 a	93.62 a	115.50 a	35.82 a	104.96 a
T_11_	2.26 c	33.81 j	2.75 b	33.22 d	45.20 d	14.09 bc	70.43 c
T_12_	1.41 e	165.07 i	1.73 f	17.12 f	16.47 g	4.00 c	20.76 f
T_13_	0.41 g	720.52 f	0.90 h	16.73 f	6.75 i	1.78 c	9.45 h
T_14_	0.13 ij	2,384.46 c	0.42 j	3.28 h	1.24 j	0.57 c	2.51 ij

The different-lettered averages in each column are significantly different according to Duncan’s multiple-range test (p ≤ 0.001). B_0_, without biochar (control); B_1_, 2.5% biochar; B_2_, 5% biochar; S_0_, 0 mM NaCl; S_1_, 25 mM NaCl; S_2_, 50 mM NaCl; S_3_, 75 mM NaCl; S_4_, 100 mM NaCl; T_0_, B_0_S_0_; T_1_, B_0_S_1_; T_2_, B_0_S_2_; T_3_, B_0_S_3_; T_4_, B_0_S_4_; T_5_, B_1_S_0_; T_6_, B_1_S_1_; T_7_, B_1_S_2_; T_8_, B_1_S_3_; T_9_, B_1_S_4_; T_10_, B_2_S_0_; T_11_, B_2_S_1_; T_12_, B_2_S_2_; T_13_, B_2_S_3_; T_14_, B_2_S_4_.

### 3.6 Leaf and root mineral content

As can be observed in **Tables 4**, **5**, the study indicates that both biochar and salt applications significantly influenced the leaf and root mineral content of pea seedlings. The mineral content in the leaves and roots of forage pea seedlings decreased, except for Na and Cl concentrations, at 25, 50, 75, and 100 mM NaCl. However, the reduction in plant mineral content under salinity stress was comparatively lower in biochar applications.

**Table 4:** The effect of biochar on the leaf mineral content of forage pea seedlings under saline conditions.

Treatments	N (%)	P (%)	K (%)	Ca (%)	Mg (%)	S (%)	Mn (mg/kg)
**T_0_** **T_1_** **T_2_** **T_3_** **T_4_**	2,27 c	0,303 c	2,23 c	0,58 b	0,100 f	0,268 b	19,32 e
	1,15 f	0,155 f	1,25 e	0,32 e	0,074 d	0,140 d	9,26 g
	0,20 h	0,038 g	0,24 g	0,07 g	0,012 f	0,037 ef	2,09 f
	0,07 ıj	0,015 hı	0,16 h	0,05 gh	0,010 f	0,015 g	1,02 ıj
	0,01 j	0,003 ı	0,05 ı	0,01 h	0,002 f	0,003 g	0,22 j
**T_5_** **T_6_** **T_7_** **T_8_** **T_9_**	2,52 b	0,330 b	3,23 a	1,04 a	0,140 b	0,283 ab	31,42 c
	1,24 ı	0,170 e	1,36 d	0,37 d	0,094 c	0,154 d	23,53 d
	0,25 h	0,041 g	0,25 g	0,08 g	0,034 e	0,032 f	16,23 f
	0,09 ı	0,015 hı	0,17 h	0,05 gh	0,009 f	0,014 g	6,13 h
	0,02 j	0,003 ı	0,04 ı	0,01 h	0,002 f	0,004 g	1,82 ı
**T_10_** **T_11_** **T_12_** **T_13_** **T_14_**	2,76 a	0,370 a	2,58 b	0,60 b	0,305 a	0,287 a	38,41 a
	1,53 d	0,197 d	1,39 d	0,49 c	0,147 b	0,205 c	33,21 b
	0,34 g	0,052 g	0,36 f	0,13 f	0,034 e	0,051 e	19,59 e
	0,12 ı	0,021 h	0,16 h	0,06 g	0,011 f	0,015 g	7,03 h
	0,02 j	0,004 ı	0,04 ı	0,02 h	0,002 f	0,003 g	1,16 ıj

	Fe (mg/kg)	Zn (mg/kg)	B (mg/kg)	Cl (mg/kg)	Na (mg/kg)	Cu (mg/kg)
**T_0_** **T_1_** **T_2_** **T_3_** **T_4_**	134,26 d	7,35 d	5,63 c	0,18 h	14,12 h	13,50 c
	101,05 e	5,52 e	4,27 e	0,53 h	206,31 gh	5,36 f
	17,40 g	1,05 g	0,82 g	1,58 g	1000,42 f	1,84 hı
	12,60 g	0,90 g	0,48 h	8,83 d	4965,38 c	0,62 ık
	2,72 h	0,21 g	0,08 ı	35,56 a	13347,48 a	0,10 k
**T_5_** **T_6_** **T_7_** **T_8_** **T_9_** **T_10_** **T_11_** **T_12_** **T_13_** **T_14_**	201,10 b	15,96 a	6,17 b	0,13 h	4,46 h	15,24 b
	137,85 d	9,45 c	4,95 d	0,41 h	84,44 h	7,46 e
	42,50 f	2,97 f	1,04 g	1,03 gh	541,05 g	2,38 h
	16,19 g	1,00 g	0,49 h	7,41 e	1931,84 e	0,87 jk
	3,54 h	0,26 g	0,20 ı	23,57 b	6606,07 b	0,18 k
	227,89 a	16,97 a	7,07 a	0,07 h	3,61 h	19,39 a
	179,72 c	11,91 b	4,79 d	0,27 h	41,07 h	9,01 d
	41,57 f	2,63 f	2,40 f	0,69 gh	207,77 gh	3,47 g
	16,75 g	1,07 g	0,83 g	3,64 f	518,77 g	1,15 ıj
	3,29 h	0,24 g	0,14 ı	13,76 c	3299,49 d	0,42 jk

The different lettered averages in each column are significantly different according to Duncan's multiple range test (p ≤ 0.001). B_0_: without biochar (Control), B_1_: 2.5% biochar and B_2_: 5% biochar, S_0_: 0 mM NaCl, S_1_: 25 mM NaCl, S_2_: 50 mM NaCl, S_3_: 75 mM NaCl and S_4_: 100 mM NaCl. T_0_: B_0_S_0_, T_1_: B_0_S_1_, T_2_: B_0_S_2_, T_3_: B_0_S_3_, T_4_: B_0_S_4_, T_5_: B_1_S_0_, T_6_: B_1_S_1_, T_7_: B_1_S_2_, T_8_: B_1_S_3_, T_9_: B_1_S_4_, T_10_: B_2_S_0_, T_11_: B_2_S_1_, T_12_: B_2_S_2_, T_13_: B_2_S_3_, T_14_: B_2_S_4._

**Table 5:** The effect of biochar on the root mineral content of forage pea seedlings under saline conditions.

Treatments	N (%)	P (%)	K (%)	Ca (%)	Mg (%)	S (%)	Mn (mg/kg)
**T_0_** **T_1_** **T_2_** **T_3_** **T_4_**	0,829 b	1,392 c	0,646 c	0,197 b	0,060 b	0,088 b	6,524 d
	0,410 f	0,734 e	0,313 f	0,110 d	0,019 e	0,046 e	3,416 e
	0,118 h	0,193 f	0,116 g	0,044 e	0,011 f	0,012 f	0,851 fg
	0,052 ı	0,075 g	0,068 ıj	0,024 fg	0,004 h	0,005 gh	0,390 gh
	0,010 j	0,015 h	0,014 l	0,005 h	0,001 ı	0,001 h	0,070 h
**T_5_** **T_6_** **T_7_** **T_8_** **T_9_**	0,950 a	1,638 a	0,748 b	0,278 a	0,065 a	0,096 a	9,600 c
	0,566 d	0,745 e	0,352 e	0,122 d	0,043 c	0,048 e	9,857 c
	0,144 g	0,181 f	0,085 hı	0,031 f	0,009 g	0,010 f	3,568 e
	0,064 ı	0,078 g	0,045 jk	0,013 gh	0,003 h	0,006 g	1,372 f
	0,011 j	0,017 h	0,029 kl	0,004 h	0,001 ı	0,001 h	0,484 gh
**T_10_** **T_11_** **T_12_** **T_13_** **T_14_**	0,684 c	1,581 b	0,845 a	0,286 a	0,064 a	0,072 c	19,516 a
	0,536 e	0,880 d	0,464 d	0,182 c	0,027 d	0,056 d	13,980 b
	0,146 g	0,210 f	0,111 gh	0,048 e	0,010 fg	0,013 f	3,532 e
	0,061 ı	0,068 g	0,049 jk	0,020 fg	0,003 h	0,004 gh	1,182 f
	0,016 j	0,016 h	0,010 l	0,005 h	0,001 ı	0,001 h	0,220 gh

	Fe (mg/kg)	Zn (mg/kg)	B (mg/kg)	Cl (mg/kg)	Na (mg/kg)	Cu (mg/kg)
**T_0_** **T_1_** **T_2_** **T_3_** **T_4_**	34,982 d	3,343 d	2,861 b	0,618 g	30,474 e	16,335 b
	16,592 f	1,712 e	0,762 f	1,709 f	215,718 e	3,039 def
	6,864 h	0,769 f	0,449 g	3,672 bc	990,318 e	1,007 fg
	2,862 ı	0,339 g	0,113 hı	3,643 bc	4097,606 c	0,274 g
	0,591 j	0,078 h	0,019 ı	4,459 a	11737,94 a	0,041 g
**T_5_** **T_6_** **T_7_** **T_8_** **T_9_**	42,007 b	5,894 a	2,334 d	0,507 g	4,793 e	8,546 c
	38,406 c	3,976 c	1,353 e	0,825 g	87,817 e	4,034 de
	9,059 g	0,979 f	0,225 h	1,501 f	475,558 e	1,098 fg
	3,628 ı	0,319 g	0,144 hı	4,031 b	1994,463 d	0,470 fg
	0,703 j	0,092 h	0,083 hı	3,478 c	7081,773 b	0,104 g
**T_10_** **T_11_** **T_12_** **T_13_** **T_14_**	68,200 a	5,093 b	3,828 a	0,496 g	8,302 e	23,324 a
	31,148 e	4,128 c	2,540 c	0,827 g	44,329 e	5,037 d
	8,500 g	0,909 f	0,645 f	1,385 f	213,482 e	1,820 efg
	3,272 ı	0,346 g	0,228 h	2,461 e	521,752 e	0,794 fg
	0,606 j	0,074 h	0,037 ı	2,900 d	7561,825 b	0,532 fg

The different lettered averages in each column are significantly different according to Duncan's multiple range test (p ≤ 0.001). B_0_: without biochar (Control), B_1_: 2.5% biochar and B_2_: 5% biochar, S_0_: 0 mM NaCl, S_1_: 25 mM NaCl, S_2_: 50 mM NaCl, S_3_: 75 mM NaCl and S_4_: 100 mM NaCl. T_0_: B_0_S_0_, T_1_: B_0_S_1_, T_2_: B_0_S_2_, T_3_: B_0_S_3_, T_4_: B_0_S_4_, T_5_: B_1_S_0_, T_6_: B_1_S_1_, T_7_: B_1_S_2_, T_8_: B_1_S_3_, T_9_: B_1_S_4_, T_10_: B_2_S_0_, T_11_: B_2_S_1_, T_12_: B_2_S_2_, T_13_: B_2_S_3_, T_14_: B_2_S_4._

## 4 Discussion

Salinity, one of the major problems in arid and semi-arid areas, adversely affects not only the morphological and physiological structure of plants but also their developmental processes (Hussain et al., 2019). Consequently, salinity significantly limits plant productivity and quality in agricultural production (Koca et al., 2007). Indeed, as observed in our study, the growth of pea seedlings was negatively affected by changes in salinity levels (**Figures 1**–**5**; **Tables 3**–**5**). The adverse impact of salinity on the growth of pea seedlings has been documented in numerous studies as well (Senturk and Sivritepe, 2015; Demirkol et al., 2019; Tetiktabanlar et al., 2020). The limitations observed in the growth of pea seedlings under salinity stress conditions may be attributed not only to the negative impact on water and osmotic pressure potential but also to a reduced carbon influx into the plant. According to the research results obtained, significant decreases were observed in SPAD, chlorophyll a, chlorophyll b, and total chlorophyll values in pea seedlings. These findings are in parallel with many studies (Yılmaz et al., 2011; Kanwal et al., 2018; Sahin et al., 2018; Zambi and Ascı, 2020).

As an abiotic factor, salinity has been found to increase the formation of reactive oxygen species (ROS) (Yan et al., 2018; Kul et al., 2021b), resulting in significant increases in H_2_O_2_ and MDA values compared to the control in our results (**Figure 4**). While ROS is a normal byproduct of plant cellular metabolism under normal conditions and serves important functions for plant growth, environmental factors such as salinity stress lead to an increase in ROS production, creating oxidative stress in plants. This, in turn, causes molecular and cellular damage resulting in cell death, significantly damaging nucleic acids and proteins and leading to lipid peroxidation in plants (Pang and Wang, 2008; Hasanuzzaman et al., 2021; Ekinci et al., 2022).

In plants, the synthesis of soluble sugars, primarily glucose and sucrose, and particularly proline, which serves as a physiological indicator of the plant’s resistance to salt stress (Liang et al., 2018), was observed to generally increase with the escalation of salinity stress in pea seedlings. Additionally, it was observed that the application of biochar led to a decrease in proline content (**Figure 4**). This situation is similar to many studies (Parida and Das, 2005; Koca et al., 2007; Mohamed et al., 2007).

When exposed to abiotic factors such as salt stress, a plant becomes highly sensitive, and significant changes occur in the hydrogen peroxide (H_2_O_2_) levels (Stone and Yang, 2006). Hydrogen peroxide, a crucial type of ROS, serves as an important signaling molecule for plant defense (Hasanuzzaman et al., 2021). In the presence of increased salt levels, even with the existence of antioxidant enzymes such as CAT, SOD, and POD in the plant, it was observed in the study that the H_2_O_2_ levels increased. This phenomenon in pea seedlings under salt stress can be explained by the plant’s antioxidant enzyme systems. An increase in salinity can enhance the activities of antioxidant enzymes such as SOD, POD, and CAT, leading to an increase in the amount of H_2_O_2_ (Duman et al., 2016).

Abscisic acid (ABA), responsible for the stimulation of genes under saline conditions, reduces the inhibitory effect of NaCl. The increase in ABA concentration in leaves under salt stress conditions, a significant abiotic factor (Zhang et al., 2006), is consistent with the results obtained in our study. In plants under salt stress, it has been determined that the levels of IAA, cytokinin, and gibberellic acid decrease, while the concentration of ABA increases (Ma et al., 2021; Ji et al., 2022; Yildirim et al., 2023). This not only supports our results but also indicates that salt stress affects water relations and membrane permeability in plant cells (Karmoker and Van Steveninck, 1979). Additionally, zeatin, an important defense mechanism of plants against drought and salinity stress (Bari and Jones, 2009; Pieterse et al., 2009), has decreased in response to the increased salinity levels (**Table 3**).

The levels of JA in pea seedlings were found to undergo changes during salt stress applications. Jasmonic acid stimulates the genes associated with protein structures that protect plants against adverse conditions, whether biotic or abiotic, enabling the activation of enzymes (Gross and Pathier, 1994). In our study, it was observed that JA decreased in the pea seedlings used due to their sensitivity to salinity (Yavas and Unay, 2018). Although salicylic acid is known to play a crucial role in the formation of systemic resistance (SAR) in plants (Metraux, 2001) and is reported to have a protective effect on plants under abiotic stress conditions, positively influencing plant growth and development in many studies conducted under salty conditions (Hao et al., 2011; Simaei et al., 2011), this positive effect can vary depending on the salt concentrations in the environment, as observed in our study (Khodary, 2004; Baran and Dogan, 2014; Souri and Tohidloo, 2019).

In plants, salinity stress can adversely affect the relationship between mineral nutrients and nutrients due to its impact on both the availability of nutrients and their translocation within the plant (Hu and Schmidhalter, 2005; Evelin et al., 2019). In this study conducted with pea seedlings, particularly the application of 100 mM NaCl resulted in lower levels of mineral nutrient elements such as N, P, K, Ca, Mg, and Fe in both leaves and roots compared to the control conditions. Additionally, it was observed that the Na and Cl levels increased more significantly compared to the control conditions (**Tables 4**, **5**). This imbalance among mineral nutrients may arise due to the competition of salinity stress with nutrient elements such as K, Ca, and NO_3_ (Hu and Schmidhalter, 2005).

Biochar is a carbon-rich material that contains organic matter and inorganic salts (humic and fulvic substances). Apart from its use for energy purposes, biochar also possesses the characteristic of being a material that can be utilized to improve soil fertility and enhance the organic carbon content of soils. Additionally, it can serve the purpose of removing heavy metals from water and soil. This dual functionality makes biochar a versatile material with applications not only in energy production but also in the enhancement of soil quality and the mitigation of heavy metal contamination in water and soil (Winsley, 2007; Lliffe, 2009). Soybean, hazelnut, tree bark, and wood pellets, including forest and agricultural residues, are used as biochar. Biochar, used to alleviate soil toxicity and reduce the adverse effects of climate change (Ennis et al., 2012; Stewart et al., 2013; Yildirim et al., 2021; Gullap et al., 2024), is also utilized in reducing the adsorption rate of sodium (Farhangi-Abriz and Ghassemi-Golezani, 2021) and mitigating the oxidative effect of NaCl (Akhtar et al., 2015). As a result of the findings obtained in our study, it has been determined that the application of biochar positively affected the growth parameters of pea seedlings (**Figures 1**–**3**). This is consistent with numerous studies (Zhang et al., 2019; Kul et al., 2021b; Ekinci et al., 2022), where biochar has been commonly used to alleviate salinity stress in plant seedlings. The application of biochar has been found to influence soil physical properties such as texture, structure, porosity, pore size distribution, available water content, and soil drainage characteristics (Glaser et al., 2002; Herath et al., 2013). These effects can further influence plant growth and development by mediating water uptake and root respiration processes. In this study, it was observed that SPAD values increased with the application of biochar. This finding may be attributed to the stimulation of plant growth, as indicated by Oktem and Oktem (2020) and Wu et al. (2023), involving plant growth-regulating effects, cellular oxidant activity, and increases in cell division and elongation. The results of this study, indicating an increase in antioxidant enzymes such as CAT, POD, and SOD, along with elevated H_2_O_2_ and MDA levels in pea seedlings due to salt stress, show similarities with previous studies (Kamran et al., 2020; Naveed et al., 2020). Consistent with our current study, it has been noted that the organic amendments used in our study were effective in alleviating the adverse effects of salt stress (Tartoura et al., 2014; Kusvuran et al., 2021). Similar to our study, the addition of biochar to the soil has been found to enhance the antioxidant enzymes and reduce the MDA content in wheat and corn plants under saline conditions (Kanwal et al., 2017; Onder and Ucar, 2021; Alamer et al., 2022; Bziouech et al., 2022).

The expression of ABA, which plays a crucial role in regulating responses to abiotic conditions such as salinity, drought, and cold stress in different plant growth processes, has been reported to increase under salt stress (Zhu, 2002; Hadiarto and Tran, 2011). This supports the results obtained in our study, as indicated by previous research (Kalifa et al., 2004; Xue and Loveridge, 2004). In the current study, it was observed that biochar, used as an organic amendment to alleviate the negative effects of salt stress, reduced the Na content in both the leaves and roots of pea seedlings. This finding suggests that the reduction in Na levels may contribute to mitigating the adverse effects of salt stress. Consequently, this reduction may lead to a decrease in the ABA hormone in pea seedlings under salt stress. Similar studies to ours have also found that biochar applications in plants have a reducing effect on Na and ABA content (Farhangi-Abriz and Torabian, 2018; Ekinci et al., 2022).

In our study, it was observed that the levels of growth hormones such as IAA, GA, SA, cytokinin, zeatin, and jasmonic acid in the leaves of pea seedlings grown under salt stress were reduced compared to the control treatment, while biochar application led to an increase in these hormones in plants (**Table 3**). Similar to the ABA enzyme, the difference observed among treatments may stem from the stress-mitigating effects of biochar applications on plants. This finding is consistent with previous studies indicating the mitigating effect of biochar on NaCl adverse impact (Thomas et al., 2013; Kim et al., 2016; Abriz and Torabian, 2018; Torabian et al., 2018).

In this study, which aimed to reduce the negative effects of salinity through biochar applications, it was observed that biochar applied at different doses reduced the levels of Na and Cl while increasing other mineral elements in the leaves and roots of pea seedlings (**Tables 4**, **5**). Biochar, which improves the physical and chemical properties of the soil and, consequently, can enhance plant productivity (Farrell et al., 2013), acts as a good nutrient carrier, reserving macro (N, P, K, Ca, etc.) and micro-nutrients (Mg, Zn, etc.) for plants (Wang et al., 2012). With a wide surface area of approximately 500 m^2^ g^-1^, biochar has been reported to increase the availability of essential nutrients in the soil, such as Ca, Mg, and K, due to its high water retention and cation exchange capacity (Lorenz and Lal, 2014). In a similar study, it was mentioned that the application of biochar, with its high adsorption capacity, reduced sodium uptake and allowed plant nutrient elements such as K, Ca, and Mg present in the soil solution to become more available due to the increase in soil water content (Akhtar et al., 2015).

In our study, when the effect of biochar obtained from olive oil pomace, which is a waste, is taken into consideration, the results obtained showed that it may have positive effects on plant development in abiotic stresses such as salinity stress. Olive oil pomace is effective in the formation of microporous structure for biochar. Moreover, the high cellulose content of olive oil pomace waste, which occurs in significant amounts in oil production, is important in the conversion of biochar by pyrolysis, which has economic value (Aissaoui et al., 2023). The use of biochar obtained from olive oil pomace production can improve the soil aggregate stability and carbon content (Kavdir et al., 2023) and increase the soil pH and organic matter content (Pellera and Gidarakos, 2015).

Re-evaluation of various wastes, especially agricultural wastes, is emphasized in order to prevent environmental pollution problems. Agricultural wastes, especially as activated carbon precursors, are an important renewable and inexpensive resource. The olive oil pomace used as biochar in this study will be important in this context. This waste as a good carbon source and amendment rich in organic matter is unfortunately not recycled in Turkey. It can be concluded from the findings of the study that biochar treatments derived from olive oil pomace improved the growth of the pea seedlings under salinity stress. Biochar, as amendment, can be used to mitigate salinity stress in agricultural lands. Olive oil pomace can be used as a source in the production of biochar.

## 5 Conclusion

Overall, pea seedlings exhibited a stress response as salinity levels rose. This included increases in hydrogen peroxide (H_2_O_2_), malondialdehyde (MDA), proline, IAA, and antioxidant enzyme activity (CAT, POD, and SOD). Conversely, there were decreases in chlorophyll content (SPAD), plant growth hormones (IAA, GA, SA, cytokinin, zeatin, and jasmonic acid), plant growth parameters, and nutrient uptake (excluding sodium and chloride). However, the study also found that applying biochar at various dosages helped to mitigate some of the negative effects of salinity stress. The findings of the study indicate that biochar amendment can enhance forage peas’ growth by modulating the plant physiology and biochemistry under salt stress. Especially 2.5% biochar application can be used to mitigate some of the negative effects of salinity stress. This suggests a potential twofold benefit: utilizing waste materials like olive oil residue for sustainable biochar production and employing the resulting biochar as a source of organic matter to enhance plant resilience against environmental stresses. Furthermore, field studies should be conducted to get information about yield.

## Data Availability

The original contributions presented in the study are included in the article/supplementary material. Further inquiries can be directed to the corresponding author.
